# Fracture of the Skull in Natural Parturitton

**Published:** 1860-06

**Authors:** 


*Fracture of the Skull in Natural Parturitton.*—M. Lize mentions, in *L'Union Medicale*, a very interesting case of a young woman, aged twenty-four, who was three days in labor, and who was delivered without instruments, after great efforts on her part. The child was dead, and the parietal bone on the left side fractured.

